# The impact of species and cell type on the nanosafety profile of iron oxide nanoparticles in neural cells

**DOI:** 10.1186/s12951-016-0220-y

**Published:** 2016-09-09

**Authors:** Freya Joris, Daniel Valdepérez, Beatriz Pelaz, Stefaan J. Soenen, Bella B. Manshian, Wolfgang J. Parak, Stefaan C. De Smedt, Koen Raemdonck

**Affiliations:** 1 Lab of General Biochemistry and Physical Pharmacy, Department of Pharmaceutics, Faculty of Pharmaceutical Sciences, Ghent University, Ottergemsesteenweg 460, 9000 Ghent, Belgium; 2 Department of Physics, Philipps University of Marburg, Renthof 7, 35037 Marburg, Germany; 3 Biomedical MRI Unit/MoSAIC, Department of Medicine, KULeuven, Herestraat 49, 3000 Louvain, Belgium

**Keywords:** Nanosafety, High content imaging, Inorganic nanoparticles, Iron oxide nanoparticles, Stem cells, Multiparametric analysis

## Abstract

**Background:**

While nanotechnology is advancing rapidly, nanosafety tends to lag behind since general mechanistic insights into cell-nanoparticle (NP) interactions remain rare. To tackle this issue, standardization of nanosafety assessment is imperative. In this regard, we believe that the cell type selection should not be overlooked since the applicability of cell lines could be questioned given their altered phenotype. Hence, we evaluated the impact of the cell type on in vitro nanosafety evaluations in a human and murine neuroblastoma cell line, neural progenitor cell line and in neural stem cells. Acute toxicity was evaluated for gold, silver and iron oxide (IO)NPs, and the latter were additionally subjected to a multiparametric analysis to assess sublethal effects.

**Results:**

The stem cells and murine neuroblastoma cell line respectively showed most and least acute cytotoxicity. Using high content imaging, we observed cell type- and species-specific responses to the IONPs on the level of reactive oxygen species production, calcium homeostasis, mitochondrial integrity and cell morphology, indicating that cellular homeostasis is impaired in distinct ways.

**Conclusions:**

Our data reveal cell type-specific toxicity profiles and demonstrate that a single cell line or toxicity end point will not provide sufficient information on in vitro nanosafety. We propose to identify a set of standard cell lines for screening purposes and to select cell types for detailed nanosafety studies based on the intended application and/or expected exposure.

**Electronic supplementary material:**

The online version of this article (doi:10.1186/s12951-016-0220-y) contains supplementary material, which is available to authorized users.

## Background

In recent years, many inorganic nanoparticles (NPs) have made their way to the market as they are being incorporated into various consumer products [1]. Moreover, their unique properties are being extensively explored for various biomedical applications. For instance, gold NPs (AuNPs) and iron oxide NPs (IONPs) hold great promise as theranostic agents for cancer treatment through hyperthermia combined with tumour detection via respectively photoacoustic or magnetic resonance imaging [2]. Additionally, silver NPs (AgNPs) are good candidates for wound dressings and antibacterial coatings of medical devices due to their enhanced antimicrobial properties [3]. However, to date only a few nano-enabled products were successfully translated into the clinic. Besides general targeting issues, this can primarily be attributed to their elusive safety profiles [4]. Despite extensive efforts, a general paradigm on how inorganic NPs are able to affect homeostasis on the level of the cell, organ or organism and to which physicochemical NP properties this can be attributed, is largely lacking [5].

In general, nanosafety evaluations struggle with two important obstacles. The first is the fast pace at which nanotechnology keeps advancing, leading to the development of a plethora of NPs with distinct physicochemical properties, which should ideally undergo safety evaluation prior to their (biomedical) implementation. The second is the lack of standardization of in vitro nanosafety studies, as various groups apply different assays on various cell types. This results in low inter-study comparability and the publication of conflicting data, which complicates the elucidation of general paradigms on NP-cell interactions [6, 7].

The first hurdle can be overcome by implementing high throughput or high content techniques in order to speed up in vitro nanosafety testing [8, 9]. Secondly, much effort is being put into the standardization of various factors of in vitro nanosafety studies [10, 11]. In this regard, we believe that the cell type selection should receive equal attention. In most studies a cell line is selected since they are in general more readily accessible, less expensive and easier to cultivate when compared to primary cells [7, 12]. However, cancer cell lines have a disturbed anti-apoptotic balance as well as an altered metabolism to sustain their high proliferation rate [13]. The phenotype expressed by immortalized cells is in turn not entirely stable and might undergo changes due to the extensive in vitro manipulation or the initial immortalization [14]. Hence, a shift towards the use of primary or stem cells as well as more complex cell culture models for in vitro nanosafety testing strategies could be noted recently. In contrast, primary cells can suffer from clonal variations and have a limited lifespan in vitro, making rational cell type selection a balancing act [7].

Subsequent to the realization that the cell type could be of substantial importance, several groups have shown that NP-induced effects vary in cell lines retrieved from different tissues or species [15–18]. On the contrary, only a few studies compared NP effects in a cancer or immortalized cell line versus primary cells representing the same tissue and species [19, 20]. Unfortunately, available data contrast one another wherefore no unambiguous conclusions could yet be formulated on whether cell lines can generally be applied as a reliable model for in vitro nanosafety studies. In addition, many of the abovementioned reports choose to either focus on interspecies variations or cell-type related differences in NP-evoked effects and do not address both factors in a single study.

Here, we present a side-by-side comparison of NP-evoked effects in six related neural cell types thereby evaluating the extent of both species and cell type related variations in NP-induced cytotoxicity. We selected a neuroblastoma cell line, neural progenitor cell line and neural stem cells derived from either humans or mice (Table 1) and purposely applied the optimal culture conditions for each cell type. These cell types were selected as potential models to assess the safety of neural stem cell labeling with nanosized contrast agents prior to transplantation in the context of regenerative medicine [21–23]. In turn, the synthesized AuNPs, AgNPs and IONPs had a diameter below 10 nm, making them good candidates for the proposed application [24]. First, we surveyed the acute toxicity of AuNPs, AgNPs and IONPs in all cell types. Subsequently we selected the IONPs for further evaluation given the minor acute toxicity. Hereto we applied a validated multiparametric approach, using automated imaging, to evaluate the effect of sublethal doses on the production of reactive oxygen species (ROS), the calcium (Ca^2+^) homeostasis, mitochondrial health and cell morphology [25]. Importantly, our data reveal distinct and cell type specific toxicity profiles that warrant careful selection of appropriate cell models for future nanosafety studies, taking both species and target tissue into account, and caution misinterpretation of experimental results based on a single cell type and/or toxicity end point.

**Table 1:** Cell types applied in this study

	Stem cells	Progenitor cell line	Cancer cell line
Human	hNSC [26]	ReNcell [27]	LA-N-2 [28]
Mouse	mNSC [26]	C17.2 [29]	Neuro-2a [30]

## Results and discussion

### Synthesized inorganic NPs display similar physicochemical characteristics

AuNP, AgNP and IONP synthesis was initiated with the aim of obtaining a similar core diameter. All NPs had a mean core diameter around 3.8 nm, as measured by transmission electron microscopy (Additional file 1: Figure S4). Subsequently all NPs were coated with poly(isobutylene-*alt*-maleic anhydride) grafted with dodecylamine (PMA), which was selected as it ensures colloidal stability over a wide pH range and a uniform coating of the different core materials [31]. Dynamic light scattering measurements in water showed a hydrodynamic diameter of 9.0, 8.9 and 12.3 nm and a negative zeta-potential around −45, −35, and −54 mV for the coated AuNPs, AgNPs and IONPs respectively. All obtained values correspond well to data reported on the characterization of NPs synthesized via similar protocols [32, 33]. The NPs were synthesized with the intention of obtaining similar physicochemical properties so that discrepancies in cell responses could be related to variations between the cell types. Additional characterization data on the plasmon resonance peaks, molecular extinction coefficients, initial NP dispersion concentrations, and electrophoretic mobility can be found in Additional file 1: Figures S5, S6 and Tables S1, S2.

### Acute toxicity depends on both the NP core material and the cell type

**Fig. 1:**
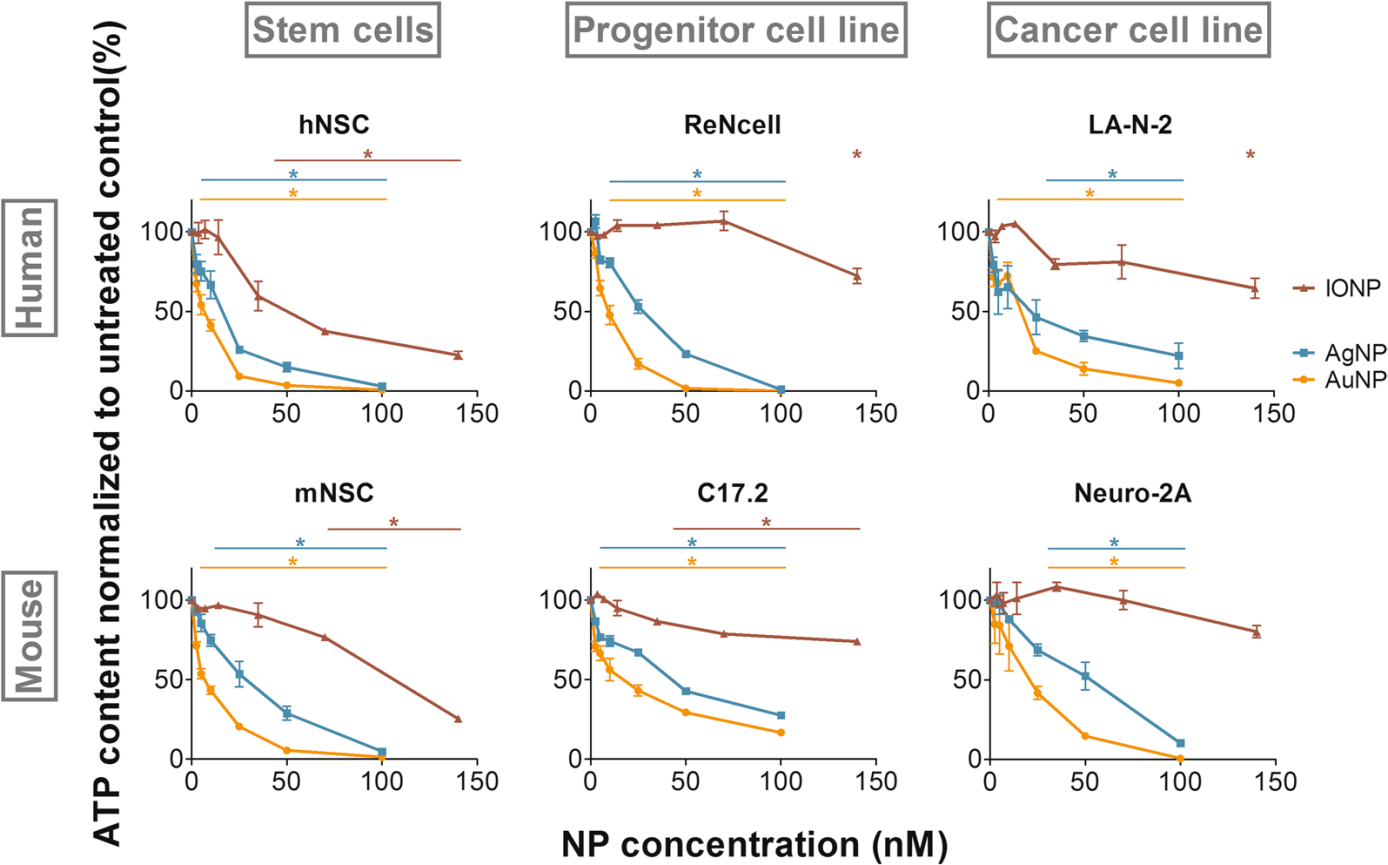
A concentration-dependent decrease in ATP content, as measured via the CellTiter GLO^®^ assay, is observed for every NP-cell type combination tested. Results for the AuNPs (*yellow*), AgNPs (*blue*) and IONPs (*red*) are represented as mean ± standard error of the mean (SEM, n = 3,). Statistical significance is indicated when appropriate for each type of NP in the corresponding color of the graphs [*p < 0.05, AuNPs (*yellow*), AgNPs (*blue*) and IONPs (*red*)]

In initial cell experiments, we evaluated cell viability following 24 h NP exposure with the CellTiter GLO^®^ assay. In Fig. 1, a general concentration-dependent decrease in ATP signal can be observed for every evaluated NP—cell type combination. Although the extent of this decrease clearly varies, the onset of this downward trend depended on both the applied NP and the cell type. In all cell types, the most severe effect was observed following AuNP treatment, while the cells were least affected by the IONPs. The toxicity observed for the AgNPs can likely in part be explained in terms of Ag^+^-ion leaching [34]. In turn, the severe acute cytotoxicity induced by the AuNPs could possibly be attributed to genotoxicity due to direct interactions between the 3.8 nm diameter AuNPs and DNA [35]. In addition, note that determining NP concentrations is not straightforward, as various methods/models need to be applied for different NP materials (Additional file 1). This may affect the comparison of absolute concentrations of NPs of different materials and may additionally explain the severe toxicity observed here for the AuNPs. Given the limited loss of cell viability observed for the IONPs, the latter were selected for further evaluation of sublethal effects.

Independent of the core material, the Neuro-2a cells were least susceptible to NP exposure whereas the hNSC, followed by the mNSC, were most sensitive. The susceptibility ranking for the other cell types varied with the NP core material. This greater sensitivity of the NSC, as found under the conditions reported here, is dissimilar to several studies where cell lines were found to be more susceptible to NP-induced acute cell injury [18, 19]. However, our data correlate well with previous work from Bregoli et al. [14] who did not observe any toxic effects in several hematopoietic cell lines, while primary bone marrow cells were clearly affected. Similar observations were recorded by Wilkinson et al. and Schlinkert et al. who independently found normal bronchial epithelial cells to experience more acute toxicity than the A549 cancer cell line [36, 37].

### ROS induction is observed in two out of six cell types

Since we found the IONPs to induce the least acute cell damage, it was decided to probe for sublethal effects caused by these NPs using a multiparametric methodology. The evaluation of effects on cell function has become crucial, as it is generally recognized that nanosafety evaluations should go beyond live/dead scoring in order to establish a more predictive paradigm [15, 38]. Subtle changes in cell function might indeed be more predictive towards in vivo adverse outcomes. For instance, NP-promoted reactive oxygen species (ROS) production in pulmonary cells has been linked to acute inflammation in the lung [39, 40]. ROS induction is also stated to be the main mechanism via which metallic NPs induce cell stress. Persisting ROS induction can subsequently lead to oxidative stress and damage cellular components such as DNA, proteins and membrane lipids [6].

**Fig. 2:**
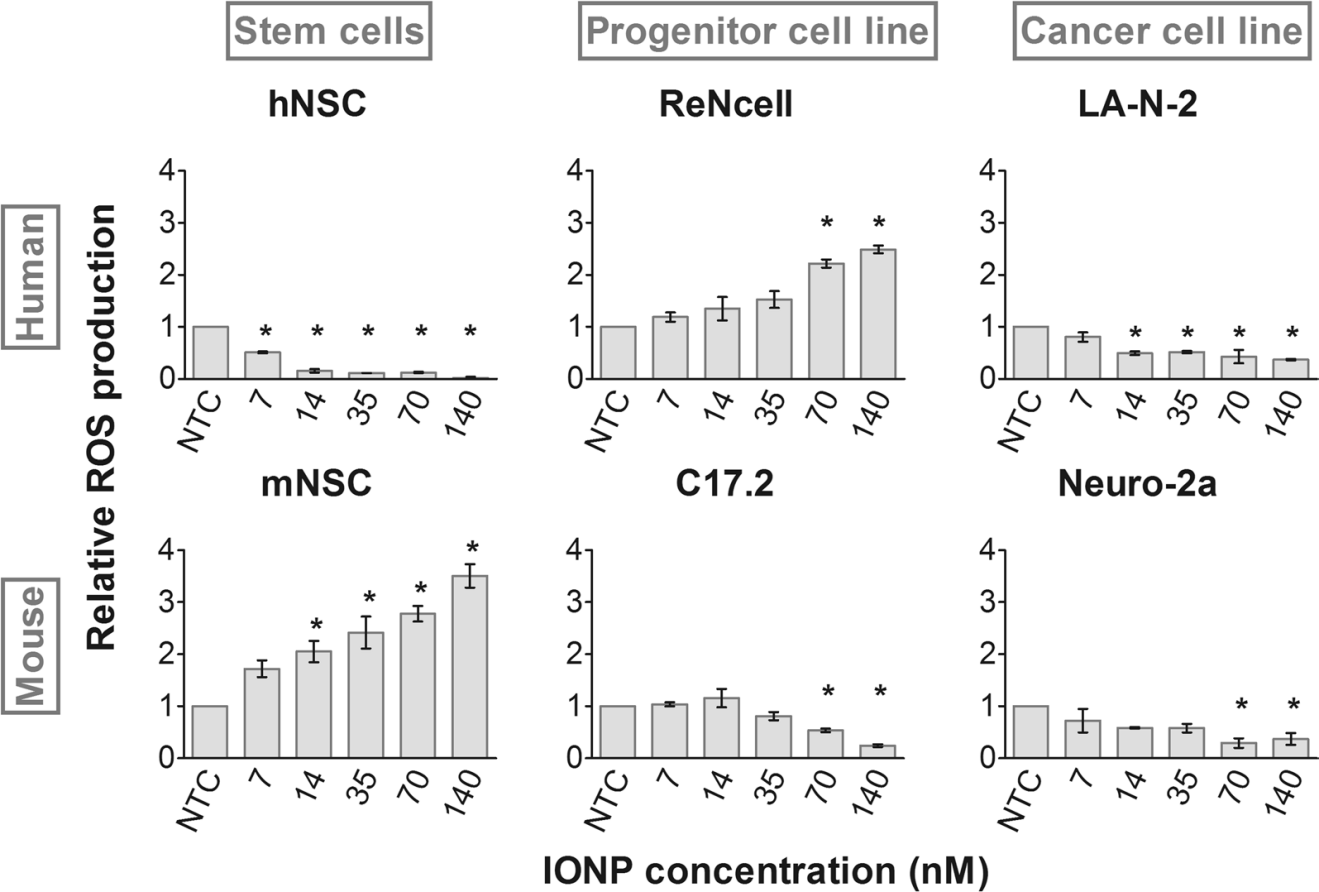
Effects on ROS production following IONP exposure visualized with the the CellROX^®^ green probe. A significant induction of ROS production was observed in the mNSC and human ReNcells. In the other four cell types a significant reduction was observed. Statistical significance is indicated when appropriate (*p < 0.05). *NTC* not treated control

Upon IONP treatment, we observed an increased ROS production in two out of six cell types, namely the mNSC and human ReNcells (Fig. 2). In all other four cell types, ROS production was significantly reduced. Notably, for both the reduced or increased ROS levels, the effect was most outspoken in the NSC. Again the murine neuroblastoma cell line (Neuro-2a) was least affected in terms of ROS. Given the variable effects, no general statements can be made on whether the human or murine cell types were more severely affected than their counterparts.

Although ROS induction by IONPs is often observed [19, 39, 41], it has been shown that NP-induced cytotoxicity cannot always be attributed to an increased ROS production [42]. Interestingly, Harris et al. also witnessed reduced ROS levels in their high content analysis of IONP-induced effects on a mammalian fibroblast cell line [6]. Additionally, IONPs can exhibit an intrinsic peroxidase-like activity in mesenchymal stem cells and thus reduce the cellular ROS content, especially of H_2_O_2_ [43, 44]. As this effect was only witnessed when IONPs remained intact, IONP biocompatibility is presumably to a large extent affected by the intracellular location and the way the cell processes the IONPs. In confirmation, Sabella et al. [45] found greater cell perturbation by metallic NPs when they were trafficked to the acidic lysosomes in comparison to the same NPs present in the cytoplasm, due to the enhanced degradation in the acidic compartments. Indeed, this degradation will be accountable for an increased amount of free iron ions, which may in turn enhance ROS production via for instance Fenton chemistry [6, 46]. A final factor that could clarify our observation is the intrinsically different anti-oxidative capacity of the various cell types [15, 17]. Thus, the cell itself likely determines NP biocompatibility to a large extent.

### IONP exposure perturbs cellular calcium homeostasis

Subsequently, we evaluated the effect of IONP exposure on the Ca^2+^ homeostasis. The intracellular free Ca^2+^ concentration ([Ca^2+^]_c_) is a valuable toxicity marker since Ca^2+^ is involved in a plethora of processes such as cell proliferation, mitochondrial function and gene expression [47, 48]. Ca^2+^ is furthermore of ultimate importance for proper cell function in neural cells, as it is required for neurotransmitter release and cellular excitability [8, 49]. Additionally, Ca^2+^ is since long known to be an important regulator of cell death, where a significant increase in [Ca^2+^]_c_ is noted [47]. A mild reduction can on the contrary be correlated with an impaired cell function due to enhanced intracellular Ca^2+^ storage or efflux in an effort to retain cell homeostasis, while cell lysis is correlated to a more severe decrease [48, 50].

**Fig. 3:**
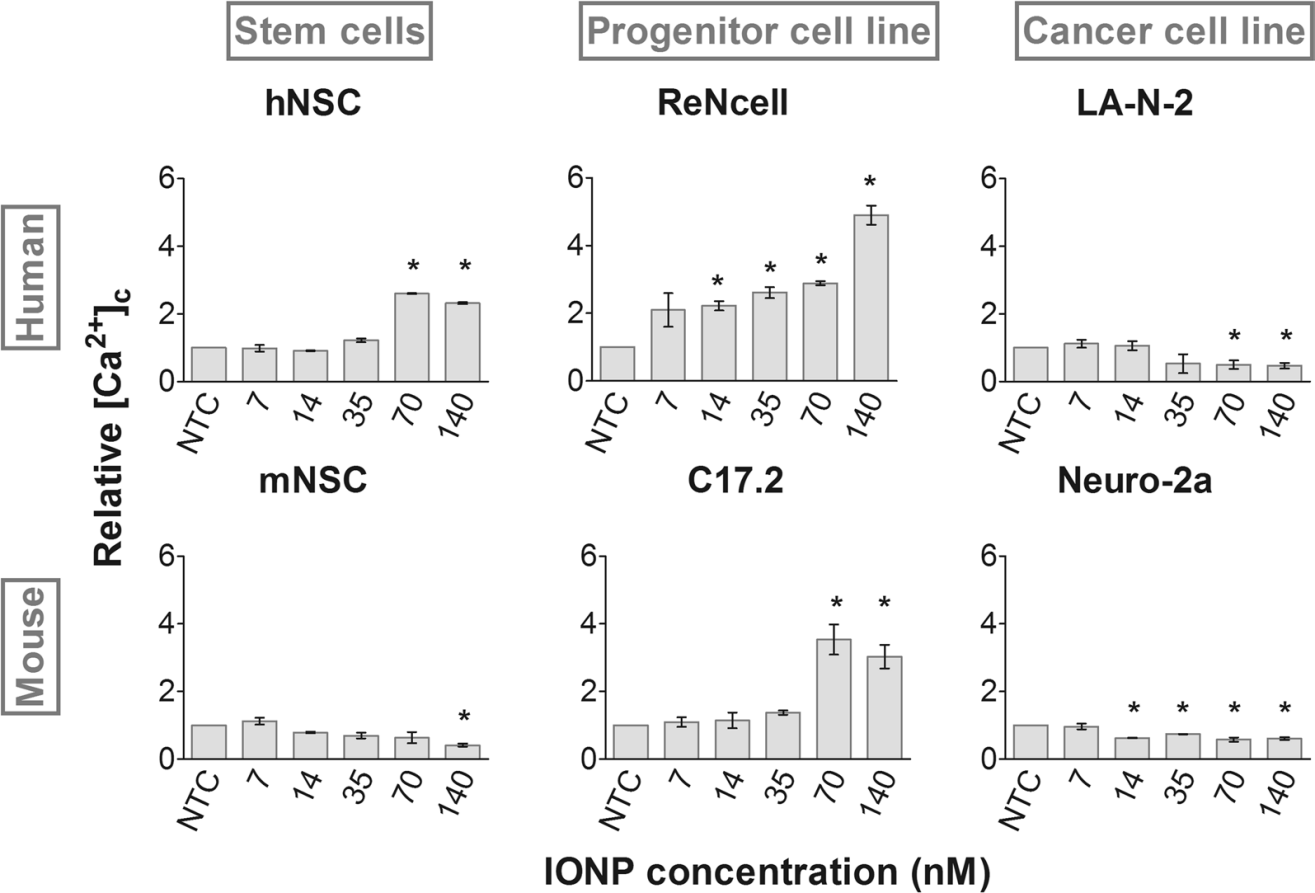
Effect on [Ca^2+^]_c_ as determined following labelling with Rhod-2 AM. A significant increase in [Ca^2+^]_c_ was observed in the hNSC and both progenitor cell lines whereas a significant reduction was observed in the remaining three cell types (p < 0.05). Statistical significance is indicated when appropriate (*p < 0.05). *NTC* not treated control

On the one hand, a significant concentration-dependent increase in [Ca^2+^]_c_ was observed in the hNSC, ReNcells, and C17.2 cells (Fig. 3). The effect was more severe in the progenitor cell lines compared to the hNSC and the ReNcells showed the highest [Ca^2+^]_c_. On the other hand, a decline of the [Ca^2+^]_c_ was detected in the mNSC, LA-N-2 and Neuro-2a cells. In contrast to previous parameters, the Neuro-2a cells showed more severe effects in terms of the perturbation of the calcium homeostasis. Again, no unambiguous conclusions could be drawn on whether human or murine cell types are more sensitive towards NP exposure.

Multiple studies investigating the influence of NP exposure on the Ca^2+^ homeostasis also found [Ca^2+^]_c_ to be augmented [51]. Since this response could be interpreted as a cell death signal, this outcome could be correlated to the initially observed acute toxicity (Fig. 1) [47, 52]. Although we would have expected to observe a greater increase in [Ca^2+^]_c_ in the hNSC when compared to the ReNcells based on the acute toxicity data, the opposite was true. In line with the observed decline in [Ca^2+^]_c_ in three out of six cell types, Haase et al. [3] documented diminished Ca^2+^ responses at cytotoxic NP doses in mNSC. This observation could on the one hand be explained in terms of cell lysis. On the other hand, stressed cells can maintain their Ca^2+^ homeostasis by elevating Ca^2+^ efflux via the plasma membrane Ca^2+^ ATPase pump [50]. We hypothesized that this occurred in the neuroblastoma cell lines where ATP levels were to a minor extent reduced and thus still allowed sufficient pump function.

In general, two groups could be distinguished based on the elevation or diminution of [Ca^2+^]_c_. Even though similar trends were retrieved in each group, it is clear that the extent of the perturbation of cellular Ca^2+^ homeostasis varied with the cell type. Since Ca^2+^ homeostasis is significantly altered upon cell transformation or immortalization in favour of cell proliferation [53], it was not surprising that NP exposure variably altered the [Ca^2+^]_c_. Notably, cell type specific toxicity profiles started to emerge as various combinations of the thus far evaluated effects were obtained.

### Mitochondria are affected by IONP loading

Next, the effect of IONP exposure on mitochondrial homeostasis was evaluated. The mitochondria are interesting organelles as they are the cell’s main energy suppliers, involved in programmed cell death, an important source of ROS and to a large extent regulated by Ca^2+^ [52, 54]. This Ca^2+^-mediated regulation is furthermore influenced by external stimuli: in combination with a stress inducer Ca^2+^ promotes ROS production and possibly cell death, whereas under physiological conditions Ca^2+^ stimulates the oxidative respiration in the mitochondria and thus ATP production [52]. Interestingly, the importance of oxidative respiration for overall cellular ATP production varies with the cell type: both cancer cells and stem cells rather rely on cytosolic glycosylation for their ATP production [54, 55]. Hence, it is conceivable that mitochondria will not only be differentially affected, but also that the impact of mitochondrial perturbation on overall cell homeostasis will vary in the different cell types.

**Fig. 4:**
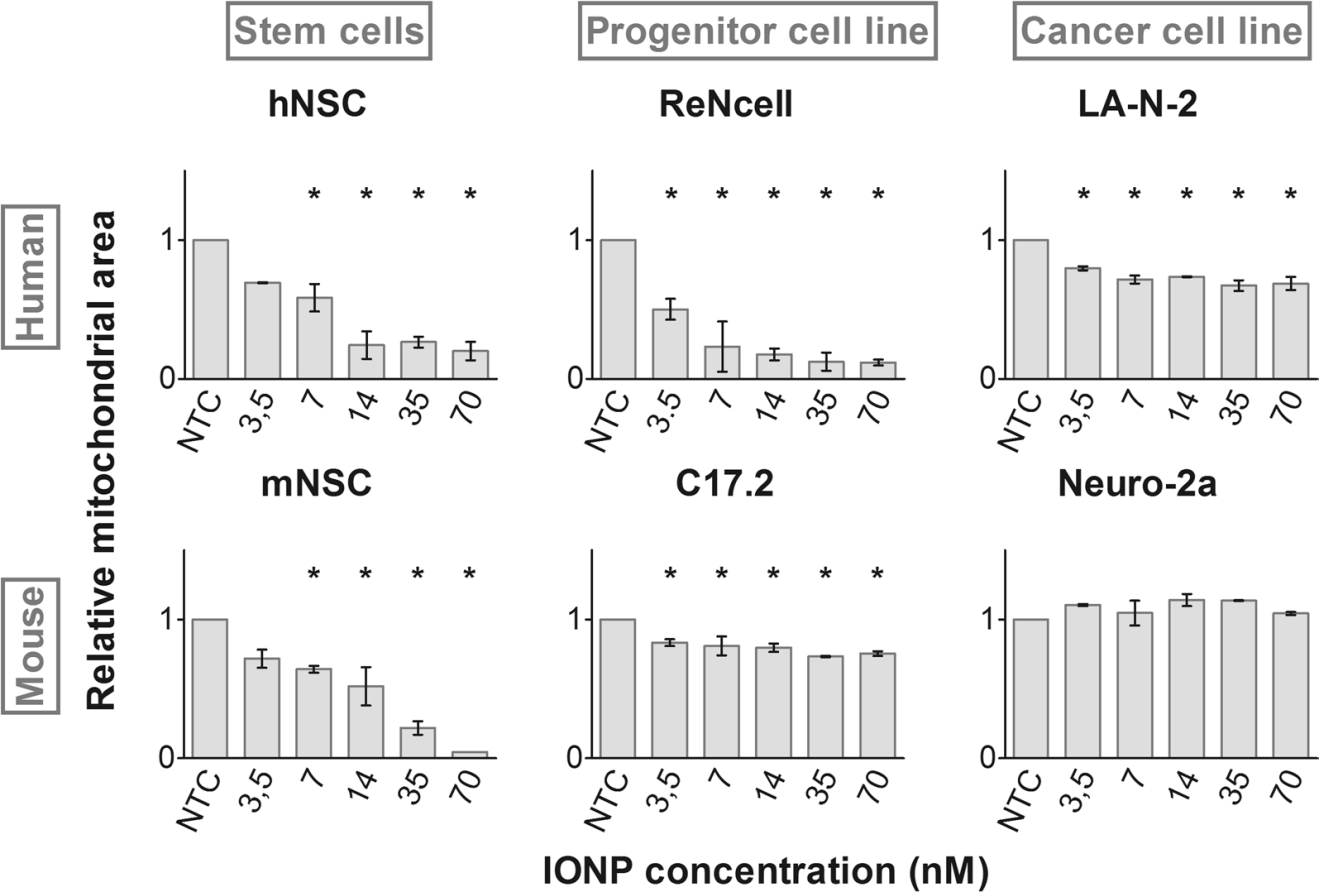
Effects on the mitochondria labelled with Mitotracker^®^ CMX-ROS in terms of the relative signal area representing the size of the mitochondrial compartment relative to the total cell area. Except for the Neuro-2a cell line, all cell types showed a significant decrease in mitochondrial area. Statistical significance is indicated when appropriate (*p < 0.05). *NTC* not treated control

To visualize the mitochondria, we selected a probe that specifically labels the organelles based on their membrane potential (ΔΨ_m_). Loss of this potential, as a result of mitochondrial membrane permeabilization, will render the organelle undetectable and has been associated with cytochrome C release and cell death initiation [52, 56]. During data analysis, such events could be detected as a reduction of the relative mitochondrial area. Figure 4 shows that all cell types, except the Neuro-2a cells, showed significant mitochondrial damage. Accordingly, the loss of ΔΨ_m_ following NP exposure has already been described in multiple studies for several NPs in cell types from various lineages and species [8, 9, 42]. In the NSC all IONP doses caused a decreased signal area, though the effect was only significant starting from 7 nM. In contrast, the affected cell lines (ReNcell, C17.2 and LA-N-2) were significantly affected by all IONP doses. The effects were most outspoken in the ReNcells, closely followed by the hNSC and mNSC. The mitochondria in the C17.2 and LA-N-2 cell lines were perturbed to a lesser extent. Notably, the human cell types were more severely affected than the murine counterpart. In addition, the neuroblastoma cell lines were most resilient on the mitochondrial level. In correspondence, Heerdt et al. [57] have previously found mitochondria in transformed cells to be less sensitive to perturbation due to an intrinsically lower mitochondrial activity and higher ΔΨ_m_.

### IONP loading affects cell morphology

Lastly, we examined alterations in cell morphology following IONP exposure. Cell morphology is a convenient parameter, especially for neural cells given their intricate architecture [8]. Moreover, numerous NPs have been shown to alter cell morphology as a secondary effect of ROS induction or via direct interactions with elements of the cytoskeleton [58, 59]. In addition to the changes in the morphological appearance, certain cell functions that require signaling via these components can subsequently be impaired [59, 60]. Thus, subtle effects on cell morphology can indirectly herald perturbation of cell function whereas severe morphological alterations, i.e. cell rounding and shrinking, can be interpreted in terms of cell death [47].

After staining the entire cell cytoplasm, the impact of IONP exposure on cell morphology was quantified via two parameters: cell area and cell circularity. The latter is applied as a measure of cell spreading and is a value between zero and one, where one represents a perfect sphere (Additional file 1). Although the extent of neurite outgrowth is often applied to evaluate the morphology of neural cells [61], this parameter was not selected for this work, as several cell types are not capable of forming neurites.

**Fig. 5:**
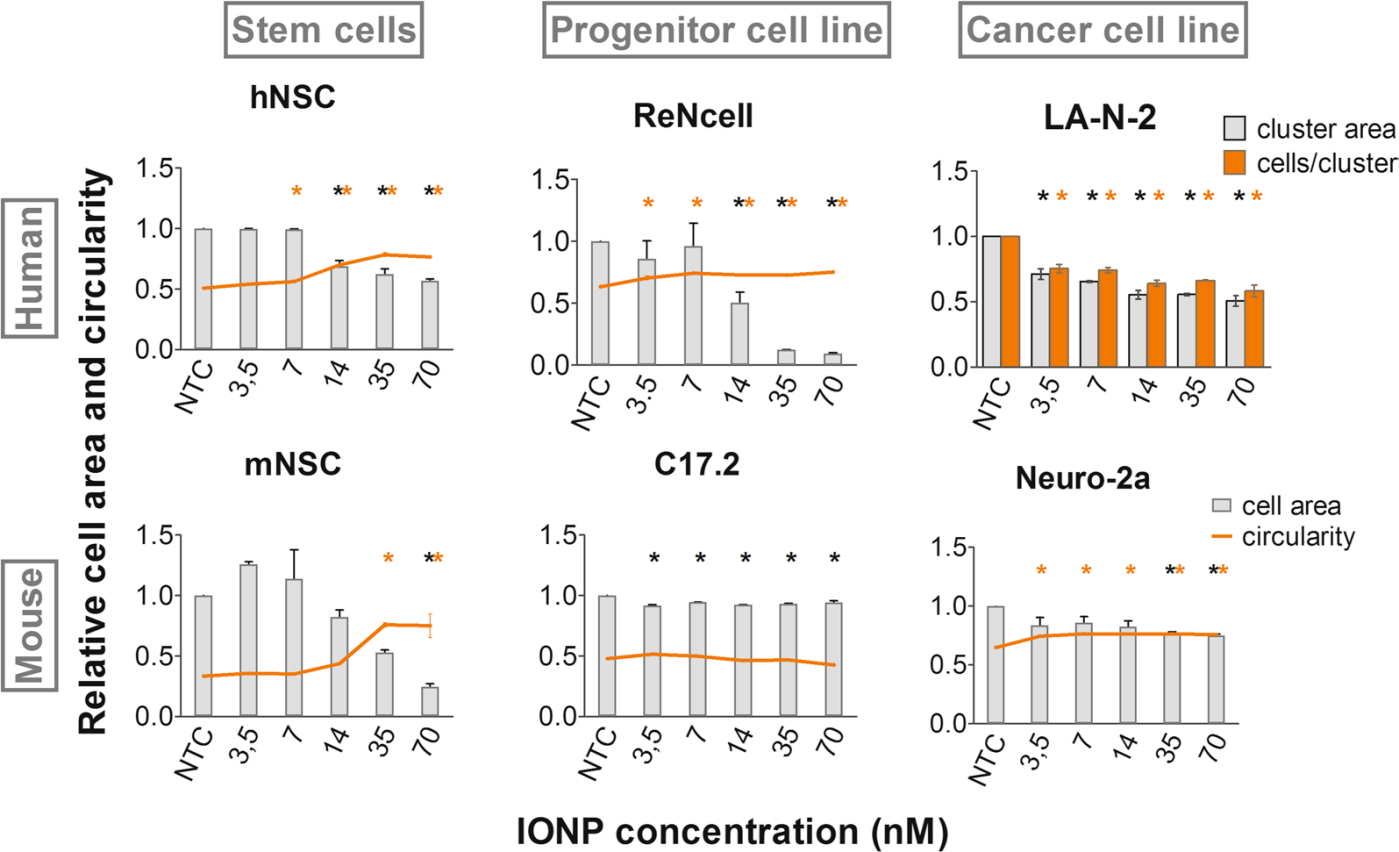
IONP-induced alterations in cell area (*grey bars*) and cell circularity (*orange lines*) visualized after labelling of the cytoplasm with the CellMask™ Blue probe for the NSC, progenitor cell lines and murine neuroblastoma cell line. Cell circularity is a measure of cell spreading and is a value between zero and one, where one represents a perfect sphere. LA-N-2 cell morphology was analysed in terms of cluster area (*grey bars*) and number of cells per cluster (*orange bars*). A decreased cell area and increased cell circularity were detected in the NSC, ReNcell and Neuro-2a cell line. For the C17.2 cells only a diminution in cell area was detected. In the LA-N-2 cell line a reduction in cells per cluster and cluster size were observed. Statistical significance is indicated when appropriate (*p < 0.05), in *black* for the cell area and *orange* in case of the cell circularity (respectively cluster area and cells per cluster in case of the LA-N-2 cell line). *NTC* not treated control

**Fig. 6:**
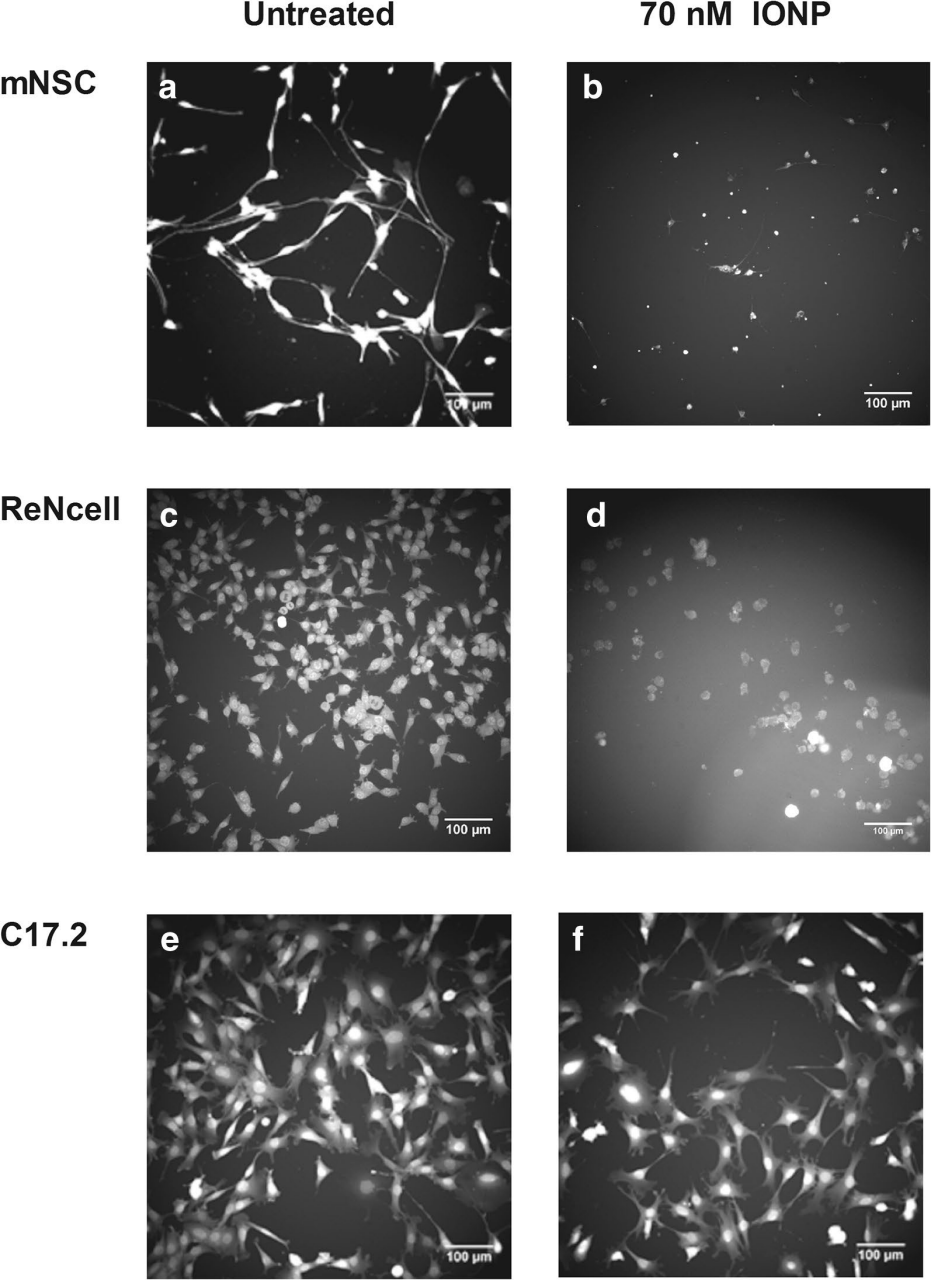
Representative images of untreated mNSCs (**a**), ReNcells (**c**) and C17.2 cells (**e**) as well exposed to 70 nM IONP (**b**, **d**, **f**). The mNSCs are affected in terms of cell area and circularity. The altered circularity in the ReNcells is less outspoken as initial morphology is less complex. Only the cell area is affected in the C17.2 cells

While only a significantly decreased cell area was noted for the C17.2 cell line, both a reduced cell area and an increase in circularity were observed in the NSC, ReNcells and Neuro-2a cells (Fig. 5; Additional file 1: Figure S8). Thus, the cells became both smaller and more spherical in a concentration-dependent fashion (Fig. 6), which was most outspoken in the ReNcells. Such loss of specific morphological features and cell shrinking has already been described in numerous studies for multiple NPs and cell types [3, 8, 22, 42]. Since it is known that cell transformation or immortalization affects cell morphology, it is not surprising that morphology was also differentially affected in the various cell types. For instance the mNSCs were more strongly affected in terms of morphology whereas only minor effects were observed in the C17.2 or Neuro-2a cell line. Since stem cells have a more intricate architecture in comparison to most cell lines, it was not surprising that the morphology of the former was impaired more extensively. Finally, as the LA-N-2 cells tend to grow in clusters we evaluated effects on cell morphology in terms of the total cluster area and number of cells per cluster, which both showed a similar concentration-dependent decrease starting from 3.5 nM IONPs. Since the decrease in cluster area was slightly more severe than the number of cells per cluster, we concluded that the cell area also decreased with every dose tested.

Overall, we observed similar effects on cell morphology (cell rounding and shrinking) in the various cell types in contrast to previously evaluated parameters. However, the exact trends and extent of the responses clearly differed. Importantly, these variations could not unequivocally be linked to one or a specific combination of responses observed for the other toxicity parameters investigated in this study, underscoring the cell type specific nature of the recorded toxicity profiles.

### Multiparametric analysis reveals cell type-specific toxicity profiles

In general, our data set reveals that each cell type reacted in a specific way to IONP exposure in terms of both extent and nature of the responses (Table 2). This could not have been deduced from the acute toxicity assessment (Fig. 1) but became increasingly clear with every additionally evaluated parameter. Furthermore, the obtained profiles would likely become increasingly complex with the addition of supplementary end points such as the influence on autophagy, induction of endoplasmic reticulum stress or genotoxicity. Note that it was not the primary objective of this study to unravel the underlying toxicity mechanisms. Hereto, additional experiments, for instance on the type of cell death or gene expression, should be performed. Instead, the aim was to clearly show the impact of both the species and the cell type, under its optimal cell culture conditions on the nanotoxicity profile within one single study. We show that for 3 different, though related neural cell types (stem cells, immortalized cells and cancer cells) the effects in the human cells were often more outspoken than the murine alternative. In addition, we found the NSC from each species to be more sensitive to IONP exposure than the cell lines.

**Table 2:** Cell type-specific nanotoxicity profiles induced by 24 h exposure to 70 nM IONPs

	ROS	Ca^2+^	Mitochondria	Cell morphology	
				Area	Circularity
hNSC	↓	↑	↓	↓	↑
mNSC	↑	↓	↓	↓	↑
ReNcell	↑	↑	↓	↓	↑
C17.2	↓	↑	↓	↓	=
LA-N-2	↓	↓	↓	/	/
Neuro-2a	↓	↓	=	↓	↑

The observed variations in cell responses can be explained in several possible ways. One may argue that variations in NP uptake in the various cell types will be an important factor. In this regard, dose heterogeneity at single cell level due to variations in NP uptake in the same population will also lead to response heterogeneity [62]. In addition, NP uptake is related to the colloidal stability in the applied cell culture media. Although we did not evaluate the abovementioned parameters in detail, it was previously shown that PMA-coated NPs show good stability in biological media and that they are taken up well by various cell types [25, 63]. Besides the extent of NP uptake, we believe that the cellular response is strongly related to the intracellular NP processing. This will in part depend on the uptake pathway since the latter will co-determine the intracellular trafficking route and the ultimate intracellular location. Indeed, as previously mentioned when NPs are present in the acidic and degrading environment of the endo-lysosomes, stronger cytotoxicity is observed than when the NPs reside in the cytosol [45]. In addition, the variations in intrinsic cell properties, such as the anti-oxidative capacity, metabolic rate (e.g. Ca^2+^ homeostasis) and mitochondrial activity, are to a large extent accountable for the revealed divergent toxicity profiles. Combined, these elements advocate an in vitro toxicity profiling that takes intrinsic cell properties and variations in the studied cell population into account. Indeed, to understand the intrinsic cellular capacity to traffic and handle exogenous materials could be of key importance to anticipate NP-evoked effects.

Furthermore, our data indicate that it is imperative to apply multiparametric methods that look beyond live/dead scoring. Notably, even when only minor variations could be detected in the cell viability, as for instance for the Neuro-2a and ReNcells, cellular homeostasis was distinctly altered. In addition, minor cell viability alterations for the ReNcells did not imply that the cell homeostasis was not impaired. Accordingly, Ge et al. [64] found IONPs to evoke important effects on cell function without affecting cell viability. Also, toxicity endpoints included in nanosafety screens should be carefully selected as some are more sensitive or indicative of the induced damage. An example of the latter is the use of cell area and circularity as parameters to describe alterations in cell morphology. Although effects on cell circularity occurred sooner, the impact on cell area was more outspoken and illustrative for the extent of the actual damage in cell types without a complex architecture. Finally, the safety of the coating should be investigated in further detail to determine its possible contribution to some of the observed effects.

Notably, we found that none of the cell types included in this work would be a suitable substitute for any other tested. In contrast, other groups did succeed in identifying a cell line alternative for primary cells based on similar cellular responses to NP exposure [12]. In such cases the use of those cell lines should be encouraged. However, the generalized use of cell lines should be approached with caution, especially when performing a detailed toxicity profiling to elucidate the mechanisms via which NPs alter cell homeostasis. Indeed, cell lines are not always ideal candidates for the analysis of cell function and may not be representative in terms of discrete cell perturbation [19]. Thus, it would be fitting to select a cell type based on the expected exposure and/or intended application of the NPs. We also propose to cautiously apply non-human cell types since we, as well as several other groups, have observed notable interspecies variations [15, 16].

For screening purposes the selection of a proper cell type is a balancing act. Indeed, primary cells can suffer from several drawbacks like an often limited availability, specific cultivation requirements, a limited life-span, and possible inter-batch and individual variations, which possibly limit the throughput [7, 12]. Hence, cell lines are still the preferred candidates when performing a large-scale screening of numerous NPs. For this reason and because it is highly unlikely that one single cell type will emerge as a universal model, we strongly believe that the definition of a set of standard cell lines would constitute a definite asset in standardizing nanosafety assessments. Additionally, the use of multiple cell types should be encouraged as it was shown to enhance the predictive power of in vitro nanosafety assessments [65]. The selected cell types would preferably be known to mimic responses observed in primary cells and would ideally be thoroughly characterized in terms of their intrinsic properties in order to enhance our understanding of the NP-induced effects.

## Conclusions

In this work, we investigated the effect of both species and cell type related variations on NP-evoked responses in six related neural cell types via a multiparametric approach. Interestingly, the observed impact on cellular health varied widely in each cell type in terms of both the nature and extent of the analyzed effects and cell type-specific nanotoxicity profiles were obtained. Hence, conclusions on the safety of a NP should preferably not be based on the evaluation of a single toxicity end point in a single cell type. We propose to rationally select a cell model based on the envisioned (biomedical) application and/or exposure scenario, especially when performing an extensive in vitro toxicity assessment with the aim of unveiling mechanisms via which the NPs inflict cell injury. Finally, with regard to standardization of in vitro nanosafety evaluations, we strongly believe that for the safety screening of large sets of nanomaterials the selection of a set of standard cell types, representing relevant target tissues, would contribute to the generation of more consistent nanosafety data.

## Methods

### NP synthesis and characterization

AuNPs, AgNPs and IONPs were synthesized and coated with the polymer poly(isobutylene-*alt*-maleic anhydride) grafted with dodecylamine (PMA), as described in Additional file 1. Following synthesis, the core diameter was measured using transmission electron microscopy. UV/Vis spectroscopy was applied to evaluate the spectral characteristics of the NPs. With the combination of UV/Vis spectroscopy and inductively coupled plasma mass spectrometry the concentrations of the dispersions were determined. Finally the hydrodynamic diameter and zeta-potential were measured using a Zetasizer Nano ZS (Malvern Instruments). Detailed information on the characterization procedures is provided in Additional file 1.

### Cell culture

All assays were performed on six neural cell types (Table 1): human and murine neural stem cells (hNSC and mNSC, Invitrogen and Millipore, Belgium), a human and mouse-derived progenitor cell line, respectively ReNcell (Millipore, Belgium) and C17.2 (Sigma, Belgium), and finally a human neuroblastoma cell line (LA-N-2, European Collection of Cell Cultures) as well as a murine counterpart (Neuro-2a, Sigma, Belgium). All cell types were cultured according to the supplier’s guidelines. Detailed information on the applied coatings and culture media compositions can be found in Additional file 1.

The cells were cultured at 37 °C in a humidified atmosphere completed with 5 % CO_2_. Cell medium was renewed every other day and cells were split after reaching 80 % confluency. Hereto, the cells were dissociated with 0.05 % trypsin–EDTA (Invitrogen, Belgium), after which the cells were centrifuged (4 min, 300 g), resuspended in fresh culture medium and seeded at appropriate densities.

### Acute toxicity

All cell types were seeded at 25,000 cells per well in opaque 96-well plates and were allowed to settle overnight. Thereafter the cells were incubated with 2.5, 5, 10, 25, 50 and 100 nM of the AuNPs and AgNPs and 3.5, 7, 14, 35, 70 and 140 nM of the IONPs during 24 h at 37 °C (5 % CO_2_). After 24 h NP incubation, the CellTiter-GLO^®^ assay (Promega, Belgium) was performed according to the manufacturer’s instructions. In short, 100 µL of the assay buffer was added to each well. Plates were shaken during 2 min after which a 10-min incubation period was respected. Finally, the signal was measured using a GloMax^®^ 96 Microplate Luminometer (Promega, Belgium). Experiments were performed in triplicate and the data are represented as the mean ± the standard error to the mean (SEM).

### High content imaging

For the multiparametric analysis, cells were seeded in 24-well plates and were allowed to attach overnight. Optimal seeding cell densities were identified for each cell type individually. The optimal seeding density was defined as the density that would result in an 80 % confluent cell layer in the untreated control at the end point of the assay. In order to preserve the cell density/cell medium volume ratio for all cell types, we varied the latter according to the optimal cell seeding density (Table 3).

**Table 3:** Seeding densities and incubation volumes per well applied in the multiparametric analysis

	hNSC	mNSC	ReNcell	C17.2	LA-N-2	Neuro-2a
Cell density	35,000	17,500	17,500	15,000	50,000	15,000
Volume (µL)	700	350	350	300	1000	300

For the evaluation of effects on ROS production and [Ca^2+^]_c_ 7, 14, 35, 70 and 140 nM IONP dispersions were applied, whereas for the effects on cell morphology and the mitochondria 3.5, 7, 14, 35 and 70 nM were tested as effects on cell function were expected to occur starting from lower NP doses. As the volume of cell medium used for incubation was adjusted according to the cell density, the NP number/volume cell medium/cell number remained equal in all high content experiments. Similar to acute toxicity experiments, the cells were incubated with the IONPs during 24 h at 37 °C in an atmosphere containing 5 % CO_2_ after which staining and analysis were performed. This set of data is presented as mean ± SEM from to independent replicates.

### Reactive oxygen species and cytoplasmic calcium levels

To allow detection of reactive oxygen species (ROS) the general ROS marker CellROX^®^ green probe (Molecular Probes, Invitrogen, Belgium) was selected. The latter was combined with the Rhod-2 AM (Molecular Probes, Invitrogen, Belgium), which becomes strongly fluorescent upon interaction with free Ca^2+^ in the cytoplasm. Following 24 h IONP incubation, the cells were labelled with both probes as described in Additional file 1.

### Effect on mitochondrial health and cell morphology

The mitochondria were labelled with Mitotracker^®^ CMX-ROS Red (Molecular Probes, Invitrogen, Belgium), which specifically accumulates in the mitochondria based on its membrane potential. To allow evaluation of cell morphology the HCS CellMask™ Blue probe (Molecular Probes, Invitrogen, Belgium) was applied. Again, cells were labeled following 24 h of IONP exposure as explained in Additional file 1.

### Statistics

Acute toxicity data are expressed as mean ± SEM (n = 3). IN Cell data are presented as mean values normalized against the untreated control ± SEM (n = 2). Statistical analysis was performed using one-way ANOVA combined with post hoc Dunnett test.

## Additional file

10.1186/s12951-016-0220-y Full methodology on the synthesis and NP-cell interaction studies as well as and additional characterization data on the different NPs.

## Abbreviations

ΔΨ_m_: mitochondrial membrane potential
[Ca^2+^]_c_: cytosolic free calcium concentration
AgNPs: silver nanoparticles
ATP: adenosine triphosphate
AuNPs: gold nanoparticles
Ca^2+^: calcium
d_c_: core diameter
HCS: high content screening
hNSC: human neural stem cell
IONPs: iron oxide nanoparticles
mNSC: murine neural stem cell
NPs: nanoparticles
NTC: not treated control
PMA: poly(isobutylene-*alt*-maleic anhydride)
ROS: reactive oxygen species
SEM: standard error of the mean
TiO_2_ NPs: titanium dioxide nanoparticles
