# Supplementary material for: The impact of species and cell type on the nanosafety profile of iron oxide nanoparticles in neural cells
Source: J Nanobiotechnology. 2016 Sep 9;14(1):69. doi: 10.1186/s12951-016-0220-y (PMC5017038; doi:10.1186/s12951-016-0220-y)
Supplement: Supplementary file 1 — 10.1186/s12951-016-0220-y Full methodology on the synthesis and NP-cell interaction studies as well as and additional characterization data on the different NPs. [file 12951_2016_220_MOESM1_ESM.docx]

The Impact of the Species and Cell Type on the Nanosafety Profile of Iron Oxide Nanoparticles in Neural Cell Types.

*Freya Joris^▲^, Daniel Valdepérez^†^, Beatriz Pelaz^†^, Stefaan J. Soenen^◊^, Bella B. Manshian^◊^, Wolfgang J. Parak^†^, Stefaan C. De Smedt^▲,1,*^, Koen Raemdonck^▲,1^*

*^▲^* Lab of General Biochemistry and Physical Pharmacy, Faculty of Pharmaceutical Sciences, Ghent University, Ottergemsesteenweg 460, B-9000 Ghent, Belgium.

^†^ Philipps University of Marburg, Department of Physics, Renthof 7, D-35037 Marburg, Germany.

*^◊^* Biomedical MRI Unit/MoSAIC, Department of Medicine, KULeuven, Herestraat 49, B-3000 Leuven, Belgium.

^1^ The authors contributed equally to this work

* Address correspondence to: [stefaan.desmedt@ugent.be](mailto:stefaan.desmedt@ugent.be)

**SUPPORTING INFORMATION**

I) Nanoparticle (NP) synthesis

I.1) AuNP synthesis

I.2) AgNP synthesis

I.3) IONP synthesis

I.4) Synthesis of the amphiphilic polymer (PMA)

I.5) Polymer coating of the NPs

I.6) Purification of the NPs

II) Nanoparticle characterization

II.1) Transmission electron microscopy (TEM)

II.2) Inductively coupled plasma mass spectrometry (ICP-MS)

II.3) UV/Vis absorption spectroscopy

II.4) Dynamic light scattering (DLS) and laser Doppler anemometry (LDA)

III) Nanoparticle-cell interactions

III.1) Cell culture

III.2) Multiparametric cytotoxicity evaluation

III.3) Reactive oxygen species (ROS) and cytoplasmic free calcium levels

III.4) Effects on mitochondria and cell morphology

IV) References

**I) Nanoparticle (NP) synthesis**

*I.1) AuNP synthesis*

The synthesis of gold nanoparticles (AuNPs) was performed according to previously reported protocols [[1](#_ENREF_1),[2](#_ENREF_2)]. First, 0.300 g of the aqueous gold precursor, chloroauric acid (HAuCl_4_, Alfa Aesar, USA), was dissolved in 35 mL Milli-Q water. 80 mL of a 27.125 mg/mL tetraoctylammonium bromide (TOAB, Sigma-Aldrich, USA) solution in toluene was added and the mixture was vigorously stirred during 5 minutes to ensure gradual transfer of the AuCl_4_^-^ ions from the aqueous to the organic phase.

$${{N\left( C_{8}H_{17} \right)}_{4}^{+}}_{organic}+{{AuCl}_{4}^{-}}_{aqueous}\underset{\to}{}{{N\left( C_{8}H_{17} \right)}_{4}^{+}{AuCl}_{4}^{-}}_{organic}$$

After carefully discarding the aqueous solution and transferring the organic phase to a 250 mL round bottom flask, 25 mL of a 13.36 mg/mL sodium borohydride (NaBH_4_, Roth, Germany) solution was added drop-wise under vigorous stirring. This caused an immediate reduction of the gold precursor, which could be observed by a color shift from light orange to dark purple.

$${{AuCl}_{4}^{-}}_{organic}\underset{\to}{+3 e^{-}}{{Au}_{organic}+ 4\cdot Cl}_{aqueous}^{-}$$

Following one hour of additional stirring to allow complete reduction of the remaining Au^+^ ions, the solution was transferred to a separation funnel. Here, the resulting AuNPs were washed under vigorous shaking with 25 mL 0.01 M HCl to remove the excess NaBH_4_. After removal of the aqueous solution the same procedure was repeated with 25 mL 0.01 M NaOH (Roth, Germany) in order to eliminate the remaining acid. Finally, the AuNPs were washed up to four times with Milli-Q water to dispose residual salts created in the previous washing steps. In the interest of obtaining a thermodynamically stable size distribution, the obtained dispersion was stirred overnight to allow the Ostwald ripening process to take place. Next, 10 mL of the surfactant 1-dodecanethiol was added and the resulting mixture was placed at 65 °C for 3 hours to allow the attachment of the 1-dodecanethiol ligand to the AuNP surface. This occurred *via* the high affinity of the thiol groups for the AuNP surface, thereby replacing the residual Br^-^ ions. Subsequently, large agglomerates were removed by centrifuging at 1000 rpm, followed by precipitation *via* methanol addition and a second centrifugation step at 1000 rpm to obtain the AuNP precipitate. This precipitate, containing dodecanethiol-coated AuNPs, was dispersed in chloroform and stored at room temperature until further use.

*I.2) AgNP synthesis*

Silver nanoparticles (AgNPs) were synthesized as previously reported [[3](#_ENREF_3),[4](#_ENREF_4)]*.* First, the stabilizer sodium S-dodecylthiosulfate, was synthesized by adding 25 mmol sodium thiosulfate pentahydrate (Sigma-Aldrich, USA) in Milli-Q water to a solution of 25 mmol 1-bromododecane (Sigma-Aldrich, USA) in 50 mL ethanol, which was prepared at 50 °C. This solution was stirred and refluxed at 50 °C for 3 hours after which it was kept at 4 °C overnight. A white precipitate was formed and isolated *via* filtration and vacuum drying.

In order to synthesize the AgNPs, 1.68 mmol AgNO_3_ (Aldrich, USA) was added to 1.26 mmol of sodium S-dodecylthiosulfate that was previously dissolved in ethanol at 50 °C. This mixture was stirred for 10 minutes, during which the solution became light brown. Next, a solution of 8.4 mmol NaBH_4_ in 15 mL ethanol was added. As the Ag^+^ ions were reduced by NaBH_4_, a coating of the dodecylthiol ligand was formed around the AgNPs, which was visibly detected by the glooming of the dispersion into a dark brown shade. After a 5 minute incubation period, 0.42 mmol ascorbic acid (Sigma-Aldrich, USA) was added and the resulting dispersion was stirred for 3 hours. Next, the sample was slowly cooled down to room temperature and the resulting AgNPs were collected *via* centrifugation (15 minutes at 3000 rpm). After being washed with Milli-Q water, ethanol and acetone, the AgNPs were dried under vacuum and conserved dried until further use.

*I.3) IONP synthesis*

Iron oxide nanoparticles (IONPs) were synthesized according to the method described by Sun *et al.* [[5](#_ENREF_5)]*.* In short, 2 mmol of the metal precursor, iron (III) acetylacetonate Fe(acac)_3_ (Sigma-Aldrich, USA), was mixed with 10 mmol 1,2-hexadecanediol (Chemos GmbH, Germany) in the presence of 6 mmol oleic acid (Sigma-Aldrich, USA), 6 mmol oleylamine (Aldrich, USA) and 20 mL phenyl ether (Sigma-Aldrich, USA). The resulting mixture was stirred in water-free and oxygen-free conditions and consequently heated up to 200 °C during 30 minutes. Next, the temperature was increased to 265 °C for an additional 30 minutes. During the heating Fe(acac)_3_ was reduced to IONPs *via* thermal decomposition, with 1,2-hexadecanediol acting as a reducing agent. Oleic acid and oleylamine were in turn applied as surfactants to stabilize the particles during the decomposition process. The resulting dispersion was slowly cooled down to room temperature and removed from the water- and oxygen-free glove box. Subsequently, 80 mL ethanol (Roth, Germany) was added, which resulted in the precipitation of a black NP powder that was separated *via* centrifugation at 4000 rpm for 40 minutes. Next, the NP powder was dissolved in 20 mL of an organic mixture consisting of hexane with 1 vol% oleic acid and 1 vol% oleylamine after which centrifugation during 30 minutes at 4000 rpm was applied to remove undispersed aggregates. Next, ethanol was added to the supernatant to precipitate the IONPs, which were concentrated in a third 30-minute centrifugation step at 4000 rpm. Finally, the resulting pellet was redispersed in hexane and stored until further use.

*I.4) Synthesis of the amphiphilic polymer (PMA)*

The amphiphilic polymer poly(isobutylene-*alt*-maleic anhydride)-graft-dodecyl (PMA), which is capable of transferring hydrophobic nanoparticles from an organic to an aqueous phase, was prepared according to a previously published protocol [[6](#_ENREF_6)]. This polymer, of which the structure is depicted in Figure S1, consists of a poly(isobutylene-*alt*-maleic anhydride) hydrophilic backbone (6 kDa/mol, 531278, Sigma-Aldrich, USA) that has been modified with hydrophobic dodecylamine chains (D222208, Sigma-Aldrich, USA), which ensure linkage to the hydrophobic NPs. The dodecylamine chains were added in an amount that would ensure covalent binding with 75% of the anhydride rings of the poly(isobutylene-*alt*-maleic anhydride) *via* its amine groups. Note that covalent linkage is assumed, but not experimentally verified.


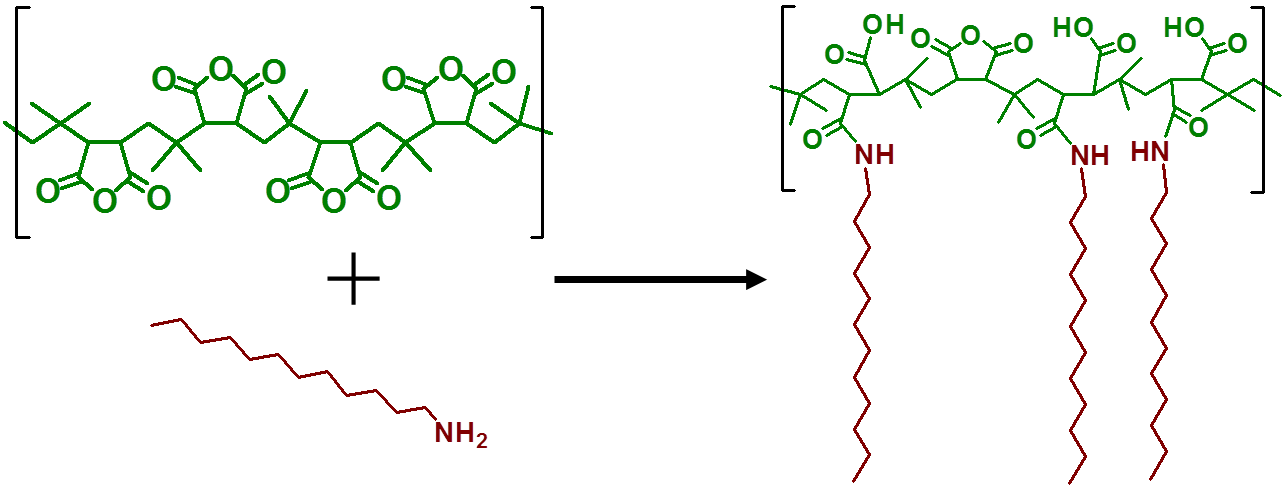


**Figure S1.** Structure of the amphiphilic poly(isobutylene-*alt*-maleic anhydride)-graft-dodecyl (PMA) with the green poly(isobutylene-*alt*-maleic anhydride) hydrophilic backbone and bordeaux hydrophobic dodecylamine chains.

In short a solution of 2.70 g (15 mmol) dodecylamine (Sigma-Aldrich, USA) in 100 mL tetrahydrofuran was added to 3.084 g (20 mmol monomer) of poly(isobutylene-*alt*-maleic anhydride) (Sigma-Aldrich, USA) powder in a round bottom flask. The resulting mixture was stirred until the solution became clear and subsequently heated to 60 °C under stirring conditions during two hours. Next, the solution was concentrated under vacuum to a final volume of approximately 40 mL, which was left at 55 °C under stirring conditions overnight. This volume decrease was necessary to allow the interaction between the anhydride rings and the amino groups. The following day the solution was dried and the powder was redispersed in 40 mL chloroform to a final concentration of 0.5 M.

*I.5) Polymer coating of the NPs*

The amount of PMA monomer required to coat the NP surface was determined *via* the following equation:

$$V_{\mathrm{polymer}} = \frac{\pi\cdot d_{\mathrm{eff}}^{2}\cdot c_{\mathrm{NP}}\cdot V_{\mathrm{NP}}\cdot R_{p/area}}{c_{\mathrm{polymer}}}$$

with d_eff_ being the effective diameter of the NP, which is defined as the sum of the NP core diameter (d_c_) as determined by transmission electron microscopy (TEM), and two times the estimated thickness of the surfactant molecule layer (l_surf_ = 1.2 nm; d_eff_ = d_c_ +2•l_surf_ ).[[6](#_ENREF_6)] c_NP_ and c_polymer_ respectively correspond to the NP and the PMA monomer concentration. Each PMA molecule comprises on average 39 monomer units. The concentration determination of the NPs is explained in detail in section II.2. V_NP_ and V_polymer_ stand for the volume of NP sample solution and polymer solution, respectively. R_p/area_ refers to the amount of PMA monomers that have to be added per effective NP surface area in monomer units per nm^2^ (in this study the value of 200 monomers PMA per nm^2^ NP surface was used, which was experimentally optimized). The calculated volume V_polymer_ was subsequently added to the preset amount of NPs in a 25 mL round bottom flask. Subsequent to the slow removal of the solvent under vacuum, 20 mL chloroform was added, which was repeated twice. After the third cycle, the dried PMA-coated NPs were dispersed in a 50 mM pH 12 sodium borate buffer (SBB 12). The opening of the anhydride rings and the origination of carboxylic groups in this basic environment ensured complete dispersion in an aqueous medium, as illustrated in Figure S2.


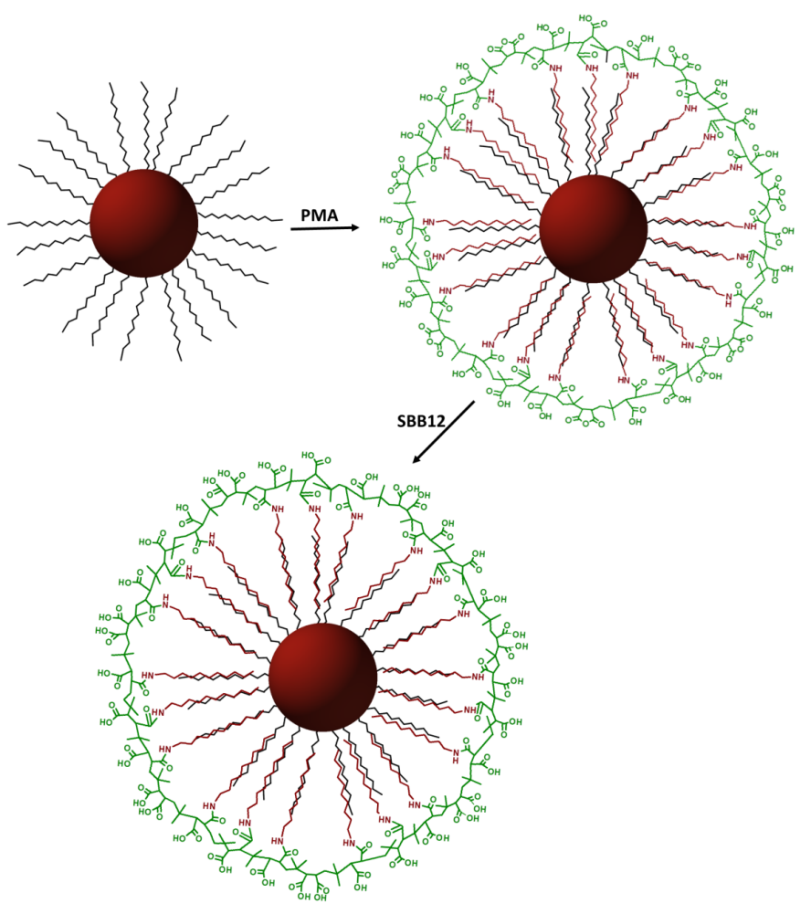


**Figure S2.** When the PMA polymer is added to the NPs, the dodecylamine chains (bordeaux) hydrophobically interact with the surfactant (black) present on the NP surface. By adding sodium borohydrate buffer of pH = 12 the anhydride rings on the poly(isobutylene-maleic acid) backbone (green) are opened and carboxylic groups are formed. It is *via* this process that the NPs acquire hydrophilic properties and are transferred to the aqueous phase [[6](#_ENREF_6)].

*I.6) Purification of the NPs*

Subsequently the aqueous PMA-coated NP dispersions were concentrated by the use of centrifuge filters (100 kDa MWCO) for 30 minutes at 3000 rpm. Then, the NPs were injected in a 2% agarose gel in a Tris-Borate-EDTA buffer (TBE 0.5x) to remove empty polymer micelles from the samples *via* gel electrophoresis [[7](#_ENREF_7)]. Before injection, a mixture of glycerol and orange G in a 1:8 proportion (v:v, glycerol + orange G solution: PMA-coated NPs) was added to improve injection into the wells of the gel (*i.e.* due the viscosity of glycerol) and to allow spotting of the front of the samples while running through the gel (*i.e.* due to the color of orange G). Once the gel was placed in the tray of the electrophoresis set-up and fully covered with TBE 0.5X, the NP samples were injected in the well and an electric field of 15 V/cm was applied during 60 minutes. The resulting NP bands on the gel (Figure S3) were cut away and transferred to a dialysis membrane (50 kDa molecular weight cut-off (MWCO)) with fresh TBE 0.5X. By applying the electric field for an additional 20 minutes the NPs were transferred out of the gel pieces into the TBE 0.5X solution. Subsequently, they were collected and washed 5 times *via* centrifugation at 3000 rpm for 20 minutes using centrifuge filters with 100 kDa MWCO membranes and Milli-Q water to remove the salts from TBE 0.5X solution. Finally, the NP dispersions were filtered with 0.2 µm syringe filter and stored in Milli-Q water until further use. Note that in our hands gel electrophoresis has so far turned out to be the best option to remove empty PMA micelles from PMA-coated NPs. However, some residual PMA micelles as impurities in the NP samples cannot be excluded.


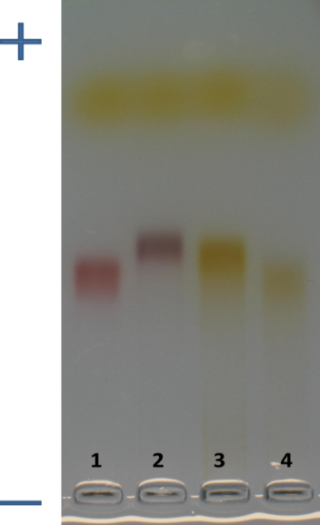


**Figure S3.** Image of a gel in which AuNPs, AgNPs and IONPs had been run with gel electrophoresis. (1) 10 nm phosphine-coated AuNPs used as a control, (2) PMA-coated AuNPs, (3) PMA-coated AgNPs and (4) PMA-coated IONPs after 1 hour gel electrophoresis in a 2% agarose gel [[8](#_ENREF_8)]. The yellow band corresponds to the loading buffer orange G used as gel front line. Due to their negative charge the NPs run towards the plus pole.

**II) Nanoparticle characterization**

*II.1) Transmission electron microscopy (TEM)*

With the aim of obtaining the inorganic core diameter (d_c_) of each batch of NPs, transmission electron microscopy (TEM) measurements were performed using a Jeol JEM3010. First, a drop of each NP sample (as dissolved in chloroform, *i.e.* before the PMA coating) was added to a TEM grid and slowly dried. Finally, pictures were recorded and analyzed using Image J to obtain a d_c_ histogram of each sample (Figure S4). For all three NP samples a mean core diameter of d_c_ = 3.8 nm was achieved. Note that core diameters with exactly the same mean value of the 3 different NP types were obtained by chance (*i.e.* our synthesis does not allow to tune the NP diameter with a tenth of nm precise) and the values have to be seen in respect to the relatively broad size distributions (Figure S4).


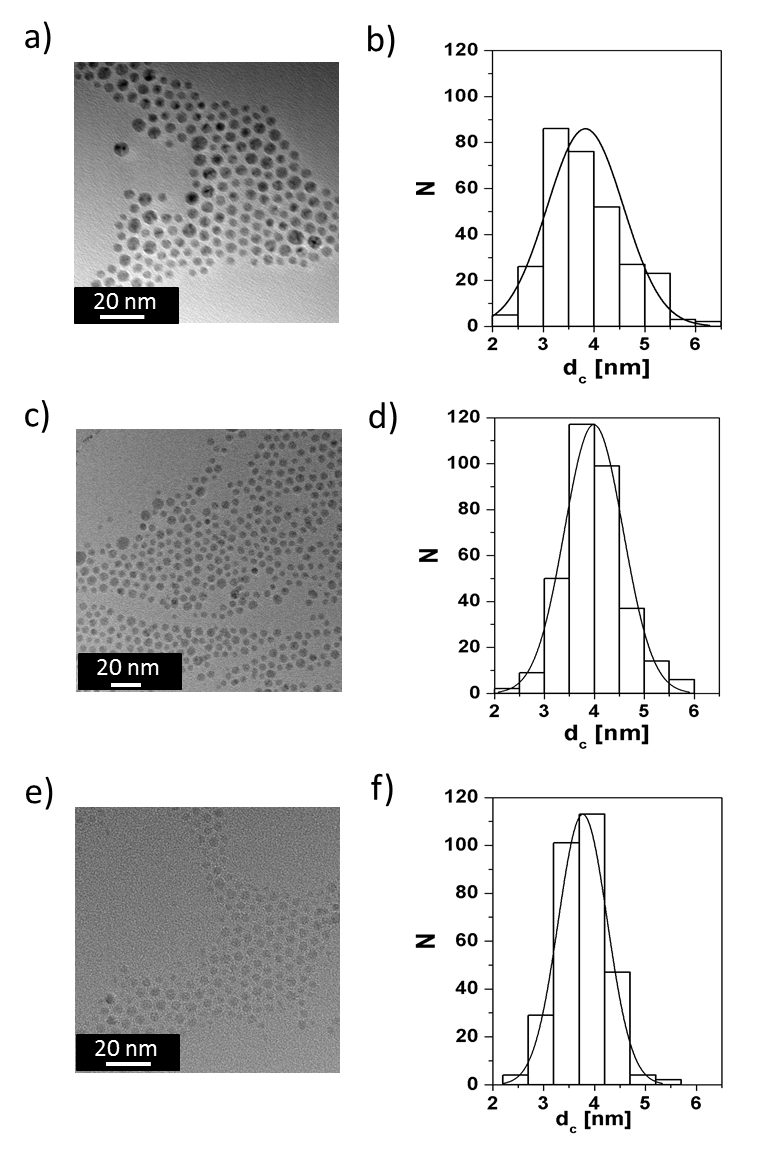


**Figure S4.** TEM images of (a) AuNPs, (c) AgNPs, and (e) IONPs, and the corresponding size distribution histograms N(d_c_) (respectively (b), (d) and (f)). For all NPs a mean d_c_ of 3.8 nm was obtained. Note that due to the width of the bars in the histograms the accuracy in the determination of the d_c_ is limited to 0.5 nm. For TEM pictures, the scale bar corresponds to 20 nm.

*II.2) Inductively coupled plasma mass spectrometry (ICP-MS)*

The concentrations of the prepared NP dispersions were measured *via* inductively coupled plasma mass spectrometry (ICP-MS, 7700 Series ICP-MS from Agilent Technologies). For concentration determination, the aqueous samples were mixed with aqua regia (1:10, V_aqua regia_ : V_NP_) and subsequently incubated at room temperature for 3 hours. These mixtures were further diluted 1:10 (V_mixture_ : V_HCl_) in 2% HCl in water and the elemental concentrations (c_M_) of the metals Au, Ag, and Fe were measured directly with ICP-MS. Subsequently the elemental metal concentrations (*i.e.* Au, Ag, or Fe) were converted into NP concentrations (c_NP_). For this purpose the mass of a single NP core (m_NP_) was first calculated *via* the equation below:

$$m_{NP}=\rho_{M}\cdot V_{c,NP}$$

in which ρ_M_ corresponds to the theoretical density of the NP core material (being 19.3 g/cm^3^ for gold; 10.49 g/cm^3^ for silver; and 5.18 g/cm^3^ for iron oxide) and V_c,NP_ refers to the volume of one NP core: V_NP_=(4/3)·π·(d_c_/2)^3^. The molar NP concentration (c_NP_) of the NP samples [mol/L] was obtained by dividing the elemental mass concentration [g/L] of the metals as obtained by ICP-MS (c_M_) by the mass of one mole of NPs (referred as the Avogadro constant, N_A_ multiplied by the mass of a single core NP, m_NP_). An additional factor F was added to the equation. For both pure metal nanoparticles (AuNPs and AgNPs) F corresponds to one. In the case of IONPs, F is the ratio of the molecular weight of iron oxide (231.53 g/mol) and the multiplication of the molecular weight of iron (55.84 g/mol) and the molar conversion factor 3 (3 moles of Fe per mol of Fe_3_O_4_). Thus for the IONPs the value of F is 1.38 (F=231.53/(55.84·3)=1.38).

$$c_{NP} = \frac{c_{M}}{m_{NP}\cdot N_{A}}\cdot F$$

The concentrations of the NP stock dispersions as determined by ICP-MS can be found in Table S1.

**Table S1. NP concentration in the initial aqueous dispersions obtained after synthesis and PMA coating as determined *via* ICP-MS measurements.**

|  | c_NP_ [μM] |
| --- | --- |
| AuNP-PMA | 1.0432 |
| AgNP-PMA | 2.0711 |
| IONP-PMA | 4.2363 |

*II.3) UV/Vis absorption spectroscopy*

UV/Vis absorption spectra were recorded with an Agilent 8453 UV-visible Spectroscopy System, which allowed the identification of the surface plasmon resonance (SPR) peaks for the AuNPs and AgNPs in Milli-Q water (cf. Figure S5). As expected, the data depicted in Figure S5 show that surface plasmon peaks were found in the AuNP and AgNP samples around 517 nm and 430 nm, respectively, while no peak was observed in the IONP absorption spectrum.

**Figure S5**. UV/Vis absorption spectra of the various NP dispersions in Milli-Q water.

For further characterization, series of minimum 4 dilutions were prepared for each NP type and analyzed with both UV/Vis absorption spectroscopy and ICP-MS, in order to experimentally determine of the extinction coefficients (ε_NP_) of the NPs (Table S2). For AuNPs and AgNPs UV/Vis absorption measurements were performed at the SPR peak wavelength, namely 517 nm and 430 nm respectively, while a wavelength of 450 nm was selected for the IONPs. The ε_NP_ were retrieved from the slopes of the linear fittings of the molar NP concentrations (c_NP_), as determined *via* ICP-MS (Table S1), and absorption values (A) measured in the same sample (with a cuvette of l = 1 cm path length). The linear fittings and ε_NP_ values can be found in Figure S6 and Table S2, respectively.

**Figure S6.** Linear fitting of NP absorbance measured at 517 nm, 430 nm, and 450 nm for the AuNPs, AgNPs, and IONPs, respectively, versus the molar concentration for different NP dispersion dilutions. According to the Lambert Beer law the slope A/(c_NP_•1cm) = ε_NP_ represents the ε_NP_ value for (a) AuNPs, (b) AgNPs, and (c) IONPs.

**Table S2: ε_NP_ values for the different NPs.**

|  | ε_NP_ [M^-1^cm^-1^] |
| --- | --- |
| AuNPs | 9510000 |
| AgNPs | 15000000 |
| IONPs | 398000 |

*II.4) Dynamic light scattering (DLS) and laser Doppler anemometry (LDA)*

The NP hydrodynamic diameter (d_h_) was measured *via* dynamic light scattering (DLS), while the zeta potential was measured *via* laser Doppler anemometry (LDA). Both measurements were performed in a Zetasizer Nano ZS (Malvern Instruments). The samples were filtered and diluted to a concentration of 10 nM in Milli-Q water. Samples were then equilibrated for 2-5 minutes at 25 °C to avoid interferences in NP movement due to temperature gradients. Measurements were performed in triplicates and the results are shown as the mean ± standard deviation (SD).

**III) Nanoparticle-cell interactions**

*III.1) Cell culture*

The human neural stem cells (hNSC, Invitrogen, Belgium) were cultured on plates coated before use with 10 µg/mL poly-L-ornithin and 6 µg/mL laminin (Sigma, Belgium) in phosphate buffered saline (PBS). Complete cell medium was obtained by supplementing KnockOutTM Dulbecco’s modified Eagle’s medium (DMEM)/F12 with StemPro® NSC FSM supplement, basic fibroblast growth factor (FGF) recombinant protein, epidermal growth factor (EGF) recombinant protein, 2 mM L-Glutamine and 2% penicillin/streptomycin (Gibco, Invitrogen, Belgium). Analogous to the hNSCs, the murine neural stem cells (mNSC, Millipore, Belgium) were cultured on plates pre-coated with 10 µg/mL poly-L-ornithin and 6 µg/mL laminin. Cell medium consisted of 2% penicillin/streptomycin containing Neural Stem Cell Expansion Medium (Millipore, Belgium) to which 20 ng/mL FGF, 20 ng/mL EGF and 2 µg/mL heparin (Millipore, Belgium) were freshly added. The human neural progenitor cell line, ReNcell VM (Millipore, Belgium), were cultivated on plates coated with 20 µg/mL laminin in DMEM/F12 (Invitrogen, Belgium) and were kept in ReNcell NSC maintenance medium (Millipore, Belgium) with 2% penicillin/streptomycin to which 20 ng/ml FGF and 20 ng/ml EGF were freshly added. For the C17.2 progenitor cell line (Sigma, Belgium) complete cell medium was prepared by adding 10% foetal bovine serum (FBS, Gibco, Invitrogen, Belgium), 5% horse serum (Gibco, Invitrogen, Belgium), 2 mM L-Glutamine and 2% penicillin/streptomycin to DMEM (Gibco, Invitrogen, Belgium). The culture medium of the human neuroblastoma cell line, LA-N-2 (European Collection of Cell Cultures) consisted of 43.5% Eagle’s minimal essential medium (EMEM, Sigma, Belgium), 43.5% HAM F12 (Sigma, Belgium), 10% FBS, 2% penicillin/streptomycin and 2 mM L-Glutamine. Finally the mouse-derived neuroblastoma Neuro-2a cell line (Sigma, Belgium) required complete cell medium consisting of 43% DMEM, 42% Opti-MEM (Gibco, Invitrogen, Belgium), 10% FBS, 2% penicillin/streptomycin and 2 mM glutamine.

*III.2) Multiparametric cytotoxicity evaluation*

An overview of the applied assays and probes can be found in Figure S7. The assays clustered in the blue box were performed using high content imaging.


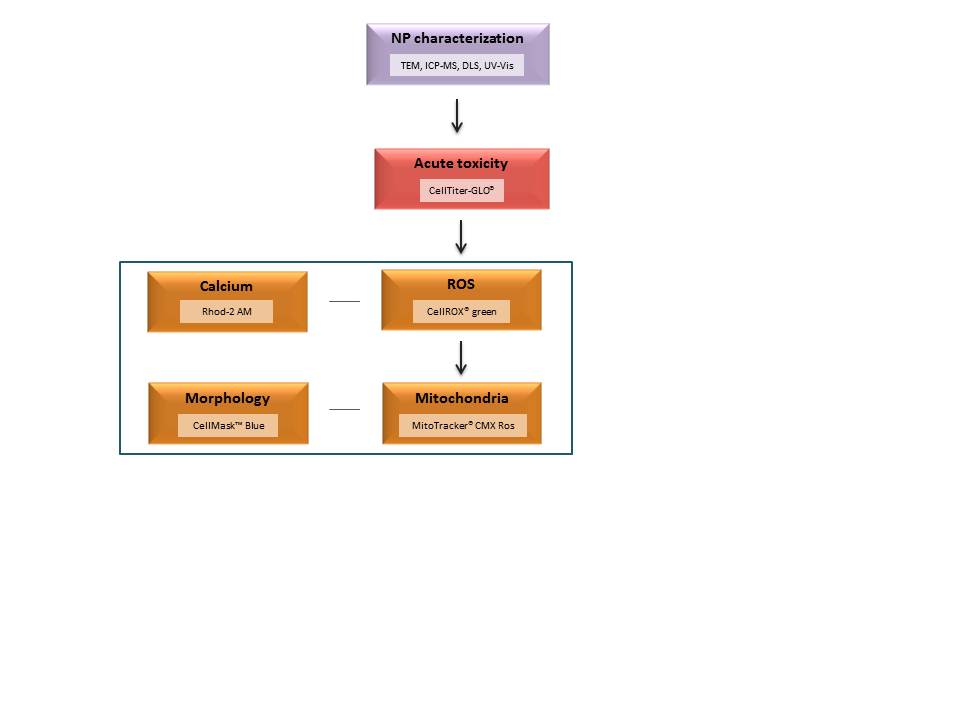


**Figure S7.** The multiparametric workflow applied for this research. The assays clustered in the box were performed with a high content imaging approach using the IN Cell Analyzer 2000.

*III.3) Reactive oxygen species (ROS) and cytoplasmic free calcium levels*

Following 24 hours exposure to the IONPs, the NP containing medium was removed. Cells were washed once with PBS with Ca^2+^ (PBS^+^, Invitrogen, Belgium) and incubated with 5 µM CellROX® green (Molecular Probes, Invitrogen, Belgium) containing cell medium (250 µL/well) during 30 minutes at 37 °C. Next, the CellROX® green containing medium was removed and cells were washed once with PBS^+^. Next a 5 µM Rhod-2 AM (Molecular Probes, Invitrogen, Belgium) solution in PBS^+^ (250 µL/well) was applied during 30 minutes at room temperature. Subsequently, the cells were washed with PBS^+^ and fixed with 4% paraformaldehyde during 15 minutes at room temperature. Finally, the fixative was removed, cells were washed once with PBS without Ca^2+^ (PBS^-^, Invitrogen, Belgium) and kept in 500 µL PBS^-^/well at 4 °C protected from light until analysis with the IN Cell Analyser 2000 (GE Healthcare Life Sciences, Belgium). ROS induction was imaged using the FITC/FITC excitation/emission filters while for the Ca2^+^ signal the Cy3/Cy3 filter combination was applied. A minimum of 60 fields was imaged per well with a 20x magnification lens resulting in minimum 5000 imaged cells per condition in each of two separate wells. Analysis of the collected images was performed with the IN Cell Developer Toolbox software (GE Healthcare Life Sciences, Belgium) using in-house developed protocols. First, the total cell number was retrieved from the FITC/FITC images by means of defining and counting the nuclei. Subsequently the basal ROS levels were determined in the control cells. To estimate the rate of ROS induction in treated wells, ROS positive cells were defined as cells with a ROS signal above background level. For all conditions the percentage of ROS positive cells was calculated by dividing the ROS positive cells by the total cell number. Finally, the percentage of ROS positive cells was normalized against the untreated control. To assess the effect on cytoplasmic free Ca^2+^, both the signal area and total cell area were determined in the Cy3/Cy3 images. Subsequently, the Ca^2+^ signal area was divided by the total cell area and the values for treated cells were normalized against the untreated control cells.

*III.4) Effects on mitochondria and cell morphology*

After 24 hours IONP incubation, the cell medium was removed and the cells were washed once with PBS^+^. Subsequently, the cells were stained with 250 µL of a 250 nM Mitotracker® CMX-ROS Red (Molecular Probes, Invitrogen, Belgium) solution in full medium to label the mitochondria based on their mitochondrial membrane potential. To this end, we respected a 30 minutes incubation period at 37 °C. Subsequently, the dye was removed and cells were washed once with PBS^+^. Next, a 4% paraformaldehyde solution was added to fix the cells (15 minutes at room temperature). Following the removal of the fixative and a single washing step using PBS^-^, we stained the cell cytoplasm using 5 µg/mL HCS CellMask™ Blue (Molecular Probes, Invitrogen, Belgium) in PBS^-^ during a 10 minute incubation period at room temperature. Finally, the HCS CellMask™ Blue solution was removed, cells were washed once with PBS^-^ and stored in 500 µl PBS^-^/well at 4°C protected from light until analysis with the IN Cell Analyser 2000. The mitochondria were optimally visualized with the TexasRed/TexasRed ex/em filter combination whereas Dapi/Dapi was applied to detect the CellMask™ Blue signal. Equal to ROS/Ca^2+^ imaging, a 20x magnification lens was applied to image at least 60 fields per well, which resulted in a minimum of 5000 cells imaged per condition in each of two separate wells. To analyze the obtained images, in-house analysis protocols were developed using the IN Cell Developer Toolbox software. First, the total cell number was retrieved from the Dapi/Dapi images by defining and counting the nuclei as HCS CellMask™ Blue preferentially locates to the nucleus. Prior to further processing in order to retrieve information on morphological parameters, cells on the border of the field were excluded from analysis. To obtain the total cell area the cells were initially segmented based on the nuclei, hereafter the cell cytoplasm was defined based on the signal intensity and eventual holes were filled. Besides the total cell area, the circularity was determined for each individual cell. This is a value between zero and one where the upper limit represents a perfect sphere. The average cell area was calculated for every condition and normalized against the untreated control. Information on the effect on mitochondrial membrane potential was retrieved from the images obtained in the orange channel. Following the definition of the mitochondria based on the signal intensity, two parameters were retrieved; namely the number of mitochondria and total area of the mitochondrial signal found within the total cell area. For both parameters the data were normalized against the untreated control cells. Sample images for the analysis of effects on cell morphology are shown in Figure S8.

|  | **Untreated** | **70 nM IONP** |
| --- | --- | --- |
|  |  |  |
| **hNSC** | 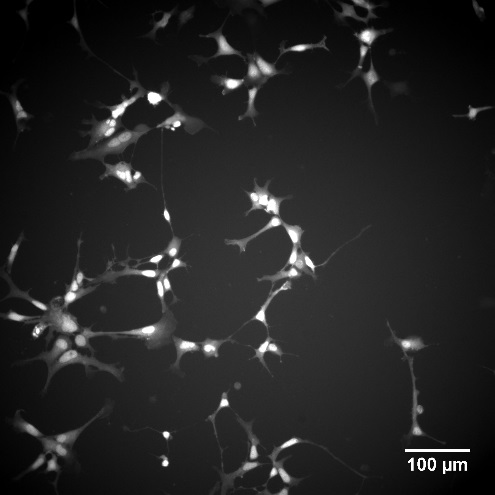 | 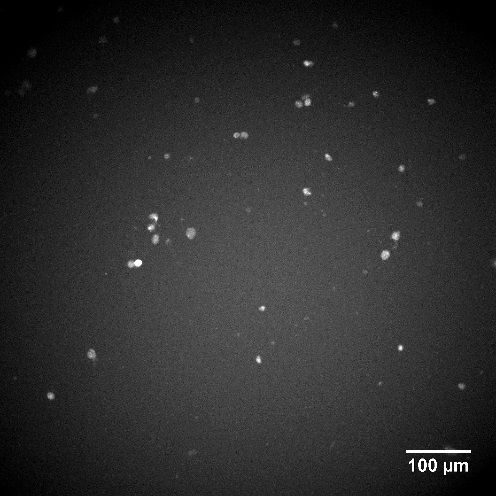 |
|  |  |  |
| **mNSC** | 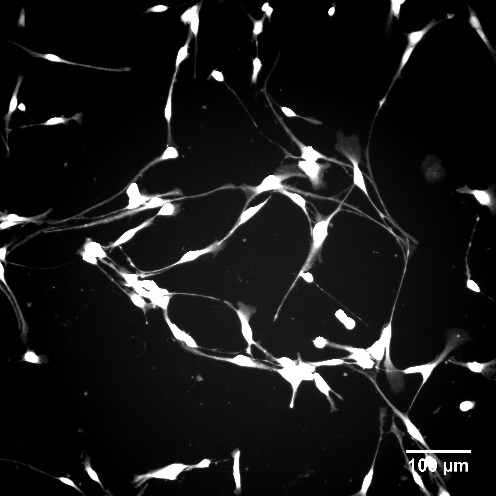 | 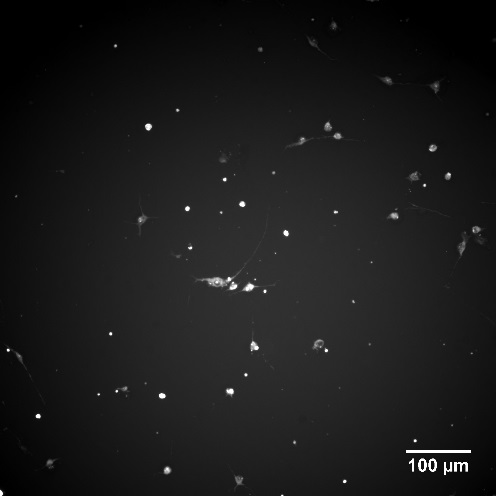 |
|  |  |  |
| **ReNcell** | 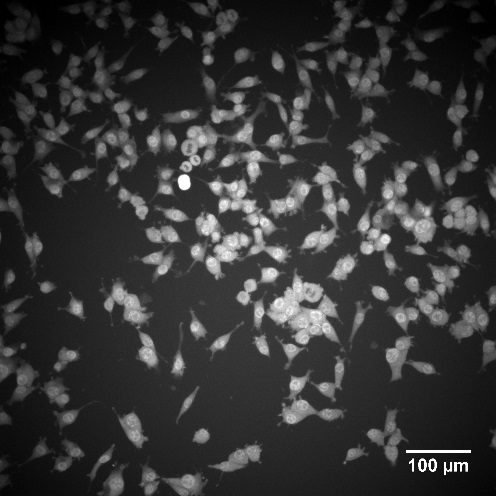 | 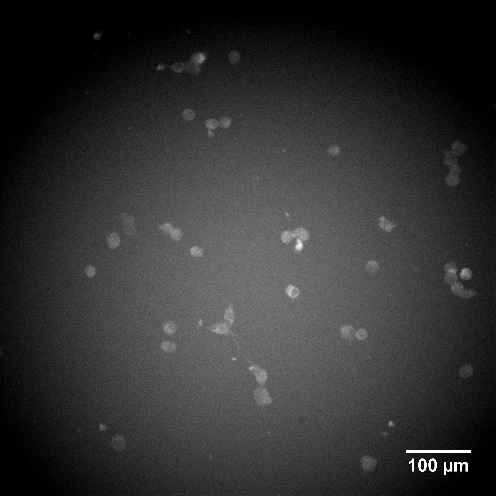 |
|  |  |  |
| **C17.2** | 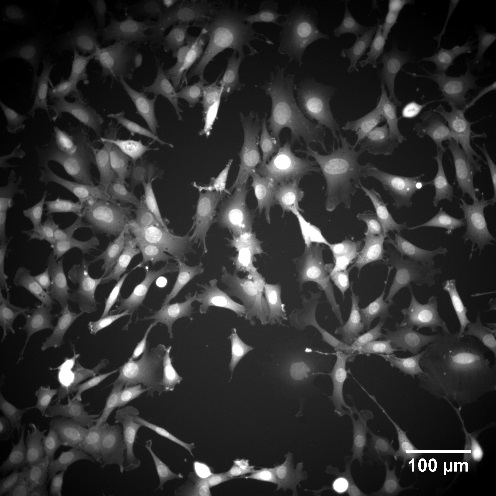 | 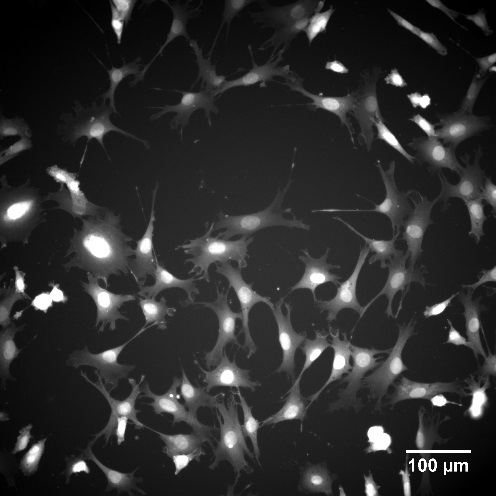 |
|  |  |  |
| **LA-N-2** | 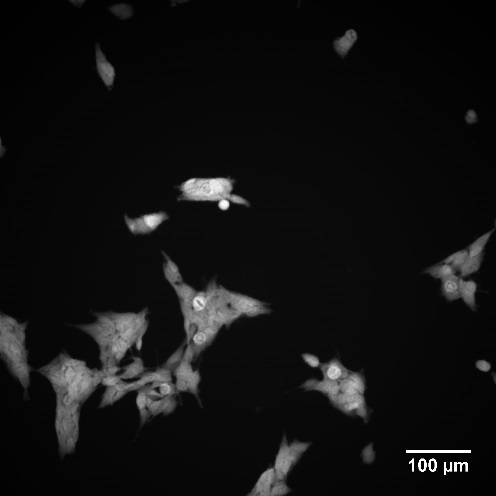 | 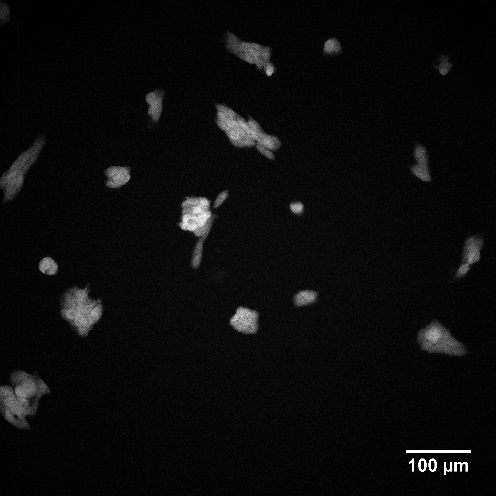 |
|  |  |  |
| **Neuro-2a** | 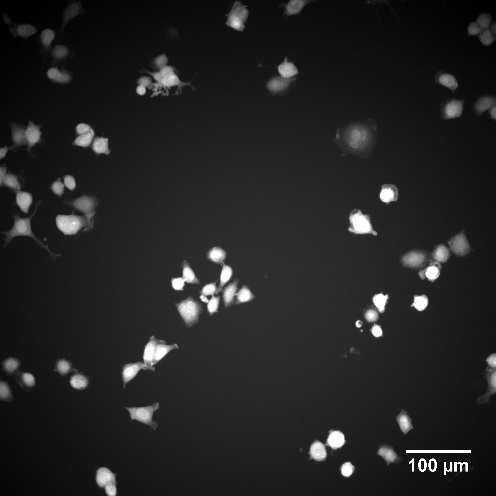 | 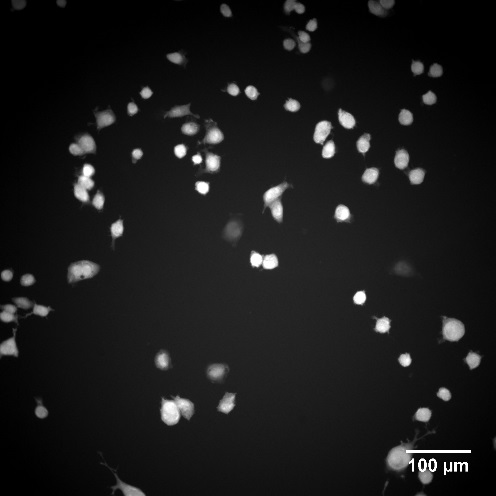 |

**Figure S8.** Representative images of IONP-induced alterations in cell morphology for the hNSC, mNSC, ReNcell, C17.2, LA-N-2 and Neuro-2a cells visualized with high content imaging after labeling the cell cytoplasm with the CellMask™ Blue probe.

**IV) References**

1. Sperling RA, Pellegrino T, Li JK, Chang WH, Parak WJ. Electrophoretic separation of nanoparticles with a discrete number of functional groups*.* *Adv Funct Mater* 16(7), 943-948 (2006).

2. Brust M, Walker M, Bethell D, Schiffrin DJ, Whyman R. Synthesis of Thiol-Derivatized Gold Nanoparticles in a 2-Phase Liquid-Liquid System*.* *J Chem Soc Chem Comm*, 801-802 (1994).

3. Mari A, Imperatori P, Marchegiani G *et al*. High Yield Synthesis of Pure Alkanethiolate-Capped Silver Nanoparticles*.* *Langmuir* 26(19), 15561-15566 (2010).

4. Caballero-Diaz E, Pfeiffer C, Kastl L *et al*. The Toxicity of Silver Nanoparticles Depends on Their Uptake by Cells and Thus on Their Surface Chemistry*.* *Part Part Syst Char* 30(12), 1079-1085 (2013).

5. Sun S, Zeng H, Robinson DB *et al*. Monodisperse MFe2O4 (M = Fe, Co, Mn) nanoparticles*.* *J Am Chem Soc* 126(1), 273-279 (2004).

6. Lin CA, Sperling RA, Li JK *et al*. Design of an amphiphilic polymer for nanoparticle coating and functionalization*.* *Small* 4(3), 334-341 (2008).

7. Fernandez-Arguelles MT, Yakovlev A, Sperling RA *et al*. Synthesis and characterization of polymer-coated quantum dots with integrated acceptor dyes as FRET-based nanoprobes*.* *Nano lett* 7(9), 2613-2617 (2007).

8. Pellegrino T, Sperling RA, Alivisatos AP, Parak WJ. Gel electrophoresis of gold-DNA nanoconjugates*.* *J Biomed Biotechnol* 2007 26796 (2007).
